# Endothelin-2 and Its Association with Uric Acid Levels and Systemic Inflammation: Relevance to Chronic Kidney Disease Progression

**DOI:** 10.3390/ijms27010540

**Published:** 2026-01-05

**Authors:** Alexander Bozhidarov Blazhev, Krasimir Kostov, Borislav Ivanov Ignatov, Tsvetelina Eftimova, Tatyana Nedkova Simeonova, Svetla Ognyanova Blazheva

**Affiliations:** 1 Department of Biology, Medical University—Pleven, 1 Kliment Ohridski Str., 5800 Pleven, Bulgaria; 2 Department of Physiology and Pathophysiology, Medical University—Pleven, 1 Kliment Ohridski Str., 5800 Pleven, Bulgaria; dr_bignatov@abv.bg (B.I.I.); tatiana.simeonova@mu-pleven.bg (T.N.S.); 3 Hemodialysis Unit, St. Marina Hospital, 5800 Pleven, Bulgaria; dr_eftimova@abv.bg; 4 Department of Clinical Laboratory, Allergology and Clinical Immunology, Medical University—Pleven, 5800 Pleven, Bulgaria; svetla.blajeva@mu-pleven.bg

**Keywords:** chronic kidney disease, endothelin-1, endothelin-2, endothelin-3, uric acid, systemic inflammation

## Abstract

Chronic kidney disease (CKD) is associated with chronic inflammation and metabolic dysregulation. While endothelin-1 (ET-1) has been extensively studied, the role of endothelin-2 (ET-2) in CKD remains poorly understood. This cross-sectional study included 76 participants, 12 healthy controls and 64 CKD patients, stratified into three groups based on estimated glomerular filtration rate (eGFR): Group 1 (eGFR ≥ 90 mL/min/1.73 m^2^), Group 2 (eGFR 45–89 mL/min/1.73 m^2^), and Group 3 (eGFR 15–44 mL/min/1.73 m^2^). Serum concentrations of ET-1, ET-2, ET-3, uric acid (UA), and inflammatory markers (hsCRP and IL-6) were measured. ET-2 levels were significantly higher in the advanced CKD group (median 24.49 pg/mL) compared to controls (median 19.32 pg/mL; *p* = 0.030). No significant differences were observed for ET-1 or ET-3 across groups. ET-2 levels positively correlated with UA (*rho* = 0.243, *p* = 0.036), hsCRP (*rho* = 0.241, *p* = 0.039), and IL-6 (*rho* = 0.244, *p* = 0.038). These findings suggest that ET-2 may represent a potential biomarker reflecting metabolic and inflammatory dysregulation in CKD and highlight its possible relevance in disease severity assessment.

## 1. Introduction

Chronic kidney disease (CKD) is a major global public health problem, affecting approximately 10–15% of the adult population. It is characterized by a progressive decline in glomerular filtration rate (GFR), leading to the accumulation of metabolic waste products, disturbances in fluid and electrolyte balance, and increased cardiovascular morbidity and mortality [1,2]. The progression of CKD is influenced by multiple interrelated mechanisms, including systemic inflammation, oxidative stress, dysregulation of mineral metabolism, and activation of the renin–angiotensin–aldosterone system [3,4,5]. Identifying reliable biomarkers that can reflect or predict the rate of CKD progression remains a critical area of research.

Endothelins (ETs) are a family of potent 21-amino acid vasoconstrictive peptides consisting of three isoforms: endothelin-1 (ET-1), endothelin-2 (ET-2), and endothelin-3 (ET-3) [6]. These peptides exert their effects primarily through two G protein–coupled receptors, endothelin type A (ETA) and endothelin type B (ETB), and are produced in various tissues, including the vascular endothelium, kidneys, lungs, and gastrointestinal tract. [7,8]. Among them, ET-1 is the most extensively studied and is known to contribute to renal vasoconstriction, sodium retention, mesangial proliferation, and inflammation. Elevated ET-1 levels have been associated with glomerular injury and progression of kidney disease [9,10].

ET-2, although structurally similar to ET-1, is less well understood in the context of kidney disease [11]. Some studies suggest that it may play a role in immune modulation and inflammation [12,13], processes that are central to CKD pathophysiology. Its expression appears to be tissue-specific and regulated by hypoxic or inflammatory stimuli [14]. The potential of ET-2 as a biomarker of CKD progression, as well as its pathophysiological relevance distinct from ET-1 and ET-3, remains to be clarified.

Uric acid (UA) is another biomolecule that has been the focus of increasing interest in the study of CKD [15]. Hyperuricemia, characterized by elevated levels of uric acid in the blood, is commonly observed in patients with reduced renal function [16]. This condition has been linked to increased oxidative stress, endothelial dysfunction, and systemic inflammation—factors that further complicate CKD [17]. The relationship between uric acid levels and endothelin signaling, particularly in relation to ET-2, is still poorly understood. However, emerging data suggest that a potential link may exist, possibly mediated by inflammatory pathways or shared regulatory mechanisms that influence both UA metabolism and endothelin expression.

In addition, systemic inflammation is a hallmark of CKD and contributes to both kidney damage and cardiovascular complications [18]. High-sensitivity C-reactive protein (hsCRP) and interleukin-6 (IL-6) are commonly used inflammatory markers that have been associated with faster decline in renal function [19,20]. Complement components such as C3 and C4 may also participate in inflammatory responses and modulate vascular injury in CKD [21,22], although their role in relation to endothelin signaling is not fully elucidated.

The aim of the present study is to investigate the associations between circulating endothelins—particularly ET-2—and uric acid levels, systemic inflammation, and other biochemical markers relevant to CKD.

## 2. Results

### 2.1. Baseline Characteristics of the Study Population

#### 2.1.1. Cohort Description

The mean age of the cohort was 62.8 ± 14.3 years, with participants ranging from 22 to 84 years. The mean age for Group 1 was found to be 42.33 ± 13.75 years, 67.84 ± 8.62 years for Group 2, and 65.59 ± 12.42 years for Group 3. Regarding sex distribution, 43% (n = 33) were male and 57% (n = 43) female. Gender distribution appeared approximately balanced across the groups and demonstrated no statistically significant differences.

#### 2.1.2. Renal Function Indicators

As renal function declined, eGFR significantly decreased from a median (Mdn) of 98 mL/min/1.73 m^2^ in Group 1 to 58 mL/min/1.73 m^2^ in Group 2 (*U* = 25, *Z* = −4.362, *p* < 0.001) and 27 mL/min/1.73 m^2^ in Group 3 (*U* = 0, *Z* = −5.063, *p* < 0.001; Table 1). Conversely, SCr significantly increased across the groups, with statistically significant differences observed between Group 1 and Group 2 (*U* = 48, *Z* = −3.738, *p* < 0.001), Group 1 and Group 3 (*U* = 0, *Z* = −5.061, *p* < 0.001), and Group 2 and Group 3 (*U* = 10, *Z* = −6.683, *p* < 0.001; Table 1).

### 2.2. Biochemical and Metabolic Markers

An analysis of calcium-phosphate metabolism was performed, which revealed that serum calcium (Ca) levels had a Mdn of 2.37 (IQR 0.16) mmol/L for the entire cohort. A pairwise comparison of Ca levels between the groups revealed no statistically significant difference.

Inorganic Pi levels were found to be parametric and are reported as mean ± SD. The independent-samples *t*-test was used for inter-group comparisons. The concentrations of Pi increased with CKD stage, from Group 1 (1.10 ± 0.17) to Group 3 (1.35 ± 0.397). Statistically significant differences were confirmed between Group 1 and Group 3 (*p* = 0.006), and between Group 2 (1.303 ± 0.254) and Group 3 (*p* = 0.018).

**Figure 1:**
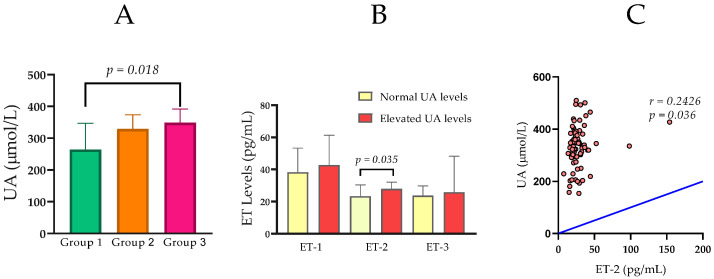
(**A**) Comparison of serum uric acid (UA) levels among Group 1 (normal renal function), Group 2 (moderately impaired renal function), and Group 3 (severely impaired renal function). (**B**) Serum ET-1, ET-2, and ET-3 levels in relation to normal and elevated levels of UA. (**C**) Statistically significant correlation between UA and ET-2 in the entire CKD cohort. Group comparisons were performed using the Mann–Whitney U test, and correlations were assessed using Spearman’s rank correlation coefficient.

UA levels increased from 264.50 (IQR 144.3) μmol/L in Group 1 to 348.50 (IQR 83.8) μmol/L in Group 3. A statistically significant difference was found between Group 1 and Group 3 (*U* = 103, *Z* = −2.345, *p* = 0.018; Figure 1A).

When the subjects were stratified according to their UA levels, those with elevated UA levels (n = 21) exhibited significantly higher concentrations of ET-2 (Mdn = 27.89 pg/mL) in comparison to those with normal UA levels (n = 55) (Mdn = 23.40 pg/mL, *U* = 396.5, *p* = 0.035; Figure 1B).

### 2.3. Inflammatory Markers

**Figure 2:**
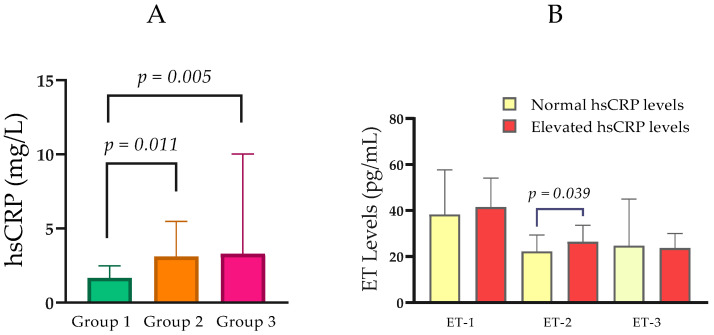
(**A**) Comparison of hsCRP levels between the groups with normal, moderately, and severely impaired renal function. (**B**) Serum ET-1, ET-2, and ET-3 levels in relation to normal and elevated hsCRP. Statistical analysis was performed using the Mann–Whitney U test; *p* < 0.05, statistically significant.

Median hsCRP concentrations increased with advancing renal dysfunction. Specifically, hsCRP was 1.68 mg/L (IQR = 1.35) in Group 1, 3.11 mg/L (IQR = 3.26) in Group 2, and 3.29 mg/L (IQR = 7.64) in Group 3. Pairwise comparisons showed that hsCRP was significantly higher in Group 2 than in Group 1 (*p* = 0.011), and in Group 3 than in Group 1 (*p* = 0.005), while there was no significant difference between Groups 2 and 3 (*p* = 0.582) (Table 1; Figure 2A).

In an analysis stratified by hsCRP status, participants with elevated hsCRP (n = 37; Mdn ET-2 = 26.40 pg/mL, IQR = 11.65) exhibited higher ET-2 concentrations than those with normal hsCRP (n = 39; median ET-2 = 22.25 pg/mL, IQR = 10.16). This difference was statistically significant (Mann–Whitney U = 523.0, *p* = 0.039; Figure 2B).

**Figure 3:**
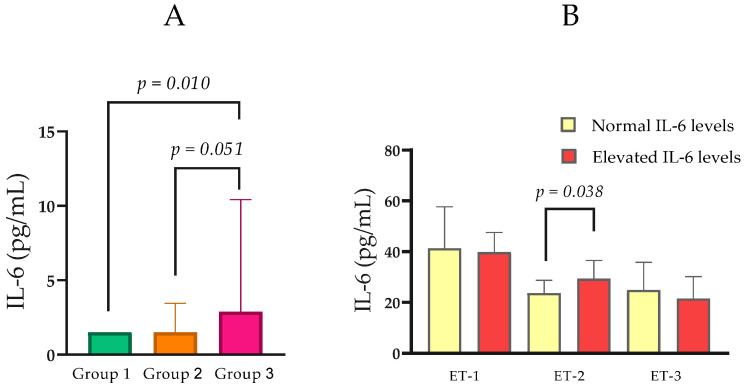
(**A**) Comparison of IL-6 levels between the groups with normal, moderately, and severely impaired renal function. (**B**) Serum ET-1, ET-2, and ET-3 levels in relation to normal and elevated IL-6. Statistical analysis was performed using the Mann–Whitney U test; *p* < 0.05, statistically significant.

Serum IL-6 concentrations varied across CKD groups. Median IL-6 was 1.50 pg/mL (IQR = 0.00) in Group 1, 1.50 pg/mL (IQR = 1.95) in Group 2, and 2.89 pg/mL (IQR = 8.92) in Group 3. Pairwise comparisons showed a statistically significant difference between Group 3 and Group 1 (Mann–Whitney *U* = 103.5, *p* = 0.018), while comparisons between Groups 1 and 2 (*p* = 0.134) and Groups 2 and 3 (*p* = 0.051) did not reach significance (Table 1; Figure 3A). In an analysis stratified by IL-6 status, participants with elevated IL-6 (n = 18; Mdn ET-2 = 29.35 pg/mL, IQR = 15.03) exhibited higher ET-2 concentrations than those with normal IL-6 (n = 58; median ET-2 = 23.67 pg/mL, IQR = 8.69; Mann–Whitney U = 352.5, *p* = 0.038; Figure 3B).

**Figure 4:**
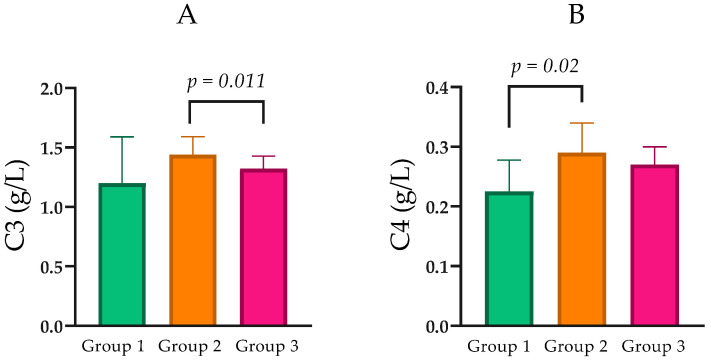
(**A**) Comparison of C3 levels between the groups with normal, moderately, and severely impaired renal function. (**B**) Comparison of C4 levels between the groups with normal, moderately, and severely impaired renal function. Statistical analysis was performed using the Mann–Whitney U test; *p* < 0.05, statistically significant.

Levels of C3 were found to be parametric and are reported as mean ± SD. A statistically significant difference was observed between Group 2 (1.44 ± 0.32 g/L) and Group 3 (1.32 ± 0.33 g/L; *p* = 0.005; Figure 4A).

Component C4 levels were analyzed as non-parametric variables across CKD groups. Median C4 was 0.23 g/L (IQR = 0.10) in Group 1, 0.29 g/L (IQR = 0.12) in Group 2, and 0.27 g/L (IQR = 0.08) in Group 3. Pairwise comparisons indicated that C4 was significantly higher in Group 2 than in Group 1 (Mann–Whitney *U* = 101.0, *p* = 0.021), while differences between Groups 1 and 3 (*p* = 0.063) and between Groups 2 and 3 (*p* = 0.436) did not reach statistical significance (Table 1; Figure 4B).

### 2.4. Endocrine Markers

Parathyroid hormone concentrations increased across CKD groups. Median PTH was 24.12 pg/mL (IQR = 15.19) in Group 1, 28.48 pg/mL (IQR = 26.20) in Group 2, and 50.68 pg/mL (IQR = 94.13) in Group 3. Pairwise comparisons indicated that PTH was significantly higher in Group 3 than in Group 1 (Mann–Whitney *U* = 49.0, *p* < 0.001) and in Group 3 than in Group 2 (Mann–Whitney *U* = 214.0, *p* < 0.001; Table 1).

### 2.5. Serum Endothelin Levels

Serum concentrations of Endothelin-1 (ET-1), Endothelin-2 (ET-2), and Endothelin-3 (ET-3) demonstrated variable trends across the study groups in relation to renal function. Given the non-parametric nature of the data, values are presented as Mdn and IQR.

**Figure 5:**
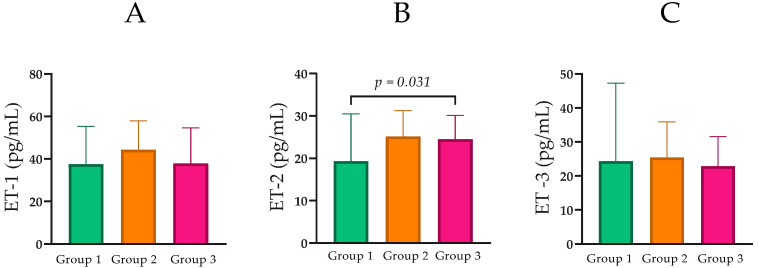
(**A**) Comparison of ET-1 levels between the groups with normal, moderately, and severely impaired renal function. (**B**) Comparison of ET-2 levels between the groups with normal, moderately, and severely impaired renal function. (**C**) Comparison of ET-3 levels between the groups with normal, moderately, and severely impaired renal function. Statistical analysis was performed using the Mann–Whitney U test; *p* < 0.05, statistically significant.

Serum ET-1 levels in Group 1 were Mdn = 37.57 pg/mL (IQR = 30.17). In Group 2, levels were Mdn = 44.32 pg/mL (IQR = 26.39). In Group 3, ET-1 concentrations were Mdn = 37.89 pg/mL (IQR = 23.46). Overall, these values show no clear pattern related to renal function, and pairwise comparisons revealed no statistically significant differences between the groups (Figure 5A).

ET-2 concentrations were compared across CKD groups. Median ET-2 was 19.32 pg/mL (IQR = 14.58) in Group 1, 25.15 pg/mL (IQR = 10.54) in Group 2, and 24.49 pg/mL (IQR = 8.62) in Group 3 (Table 1; Figure 5B). Pairwise comparisons showed a statistically significant difference between Group 1 and Group 3 (Mann–Whitney *U* = 110.5, *p* = 0.030), while differences between Groups 1 and 2 (*p* = 0.067) and Groups 2 and 3 (*p* = 0.778) were not statistically significant.

ET-3 concentrations were also analyzed across CKD groups. Median ET-3 was 24.35 pg/mL (IQR = 27.69) in Group 1, 25.43 pg/mL (IQR = 16.14) in Group 2, and 22.90 pg/mL (IQR = 13.50) in Group 3 (Table 1; Figure 5C). No pairwise comparisons for ET-3 reached statistical significance.

### 2.6. Correlations

#### 2.6.1. Relationships Between Key Clinical and Biochemical Parameters in the Entire Cohort (n = 76)

Spearman’s rank correlation was used to assess associations between clinical and biochemical parameters. In Table 2, the meaningful relationships among the variables analyzed are shown.

A strong negative correlation was found between eGFR and SCr (*rho* = −0.956, *p* < 0.001), which is expected and confirms the inverse relationship between these two markers of renal function. eGFR also exhibited a significant negative correlation with UA (*rho* = −0.378, *p* < 0.001), suggesting that declining renal function is associated with higher UA levels. Conversely, SCr showed a significant positive correlation with UA (*rho* = 0.379, *p* < 0.001).

Of particular interest to this study, UA demonstrated a statistically significant positive correlation with ET-2 (*rho* = 0.243, *p* = 0.036), indicating a potential link between elevated uric acid levels and increased ET-2 in the overall cohort (Figure 1C).

Regarding the endothelins, no statistically significant correlations were observed between eGFR and ET-1 (*rho* = −0.184, *p* = 0.117) or eGFR and ET-2 (*rho* = −0.141, *p* = 0.227). However, a significant positive correlation was found between ET-1 and ET-3 (*rho* = 0.316, *p* = 0.005). Interestingly, C4 showed a significant negative correlation with ET-1 (*rho* = −0.409, *p* < 0.001).

Furthermore, significant correlations were observed between markers of renal dysfunction and other parameters, including a negative correlation between eGFR and Pi (*rho* = −0.389, *p* < 0.001), PTH (*rho* = −0.571, *p* < 0.001), hsCRP (*rho* = −0.323, *p* = 0.005), and IL-6 (*rho* = −0.381, *p* < 0.001), and a positive correlation between SCr and Pi (*rho* = 0.307, *p* = 0.007), PTH (*rho* = 0.529, *p* < 0.001), hsCRP (*rho* = 0.270, *p* = 0.019), and IL-6 (*rho* = 0.336, *p* = 0.003). These findings underscore the complex interplay of factors associated with declining renal function.

#### 2.6.2. Correlations Within the Groups with Renal Dysfunction

To further investigate the relationships between these parameters in the context of different stages of renal dysfunction, Spearman’s Rho correlation analyses were performed separately for Group 2 and Group 3. Statistically significant correlations (*p* < 0.05) are presented below in Table 3.

In Group 2 (moderately impaired renal function), no significant correlations emerged between eGFR and UA or the endothelins. However, a significant negative correlation was noted between PTH and ET-1 (*rho* = −0.367, *p* = 0.042). Strong positive correlations were observed among several inflammatory markers, such as hsCRP with IL-6 (*rho* = 0.635, *p* < 0.001) and C3 (*rho* = 0.610, *p* < 0.001). Interestingly, C4 showed a positive correlation with ET-2 (*rho* = 0.373, *p* = 0.039) in this group.

In Group 3 (severely impaired renal function), the strong inverse relationship between eGFR and serum creatinine persisted (*rho* = −0.925, *p* < 0.001). eGFR also showed significant negative correlations with phosphate (*rho* = −0.656, *p* < 0.001) and PTH (*rho* = −0.474, *p* = 0.006). Notably, UA demonstrated a positive correlation with phosphate (*rho* = 0.434, *p* = 0.013) in this advanced stage of CKD. A positive correlation between Gender and ET-2 was observed in this group (*rho* = 0.435, *p* = 0.013). Furthermore, a significant negative correlation was found between C4 and ET-1 (*rho* = −0.586, *p* < 0.001), and a positive correlation between ET-1 and ET-3 (*rho* = 0.519, *p* = 0.002). Strong positive correlations among inflammatory markers, including hsCRP with IL-6 (*rho* = 0.723, *p* < 0.001) and C4 (*rho* = 0.696, *p* < 0.001), remained prominent.

## 3. Discussion

### 3.1. Differential Role of ET-2 Versus ET-1 in Renal Dysfunction

Our data demonstrate that, as expected, the progression of CKD is associated with a significant decrease in the eGFR and an increase in SCr, along with elevated levels of UA, PTH, and pro-inflammatory markers such as hsCRP and IL-6 [23,24]. The main and most distinctive finding of this study is the establishment of a specific trend for increasing ET-2 levels with advancing renal dysfunction.

In contrast to ET-1, ET-2 concentrations were found to be higher in patients with CKD compared to healthy controls. Specifically, the Mdn ET-2 levels were 19.32 pg/mL (IQR = 14.58) in the control group (Group 1), rising to 25.15 pg/mL (IQR = 10.54) in patients with moderate dysfunction (Group 2), and 24.49 pg/mL (IQR = 8.62) in the severe renal failure group (Group 3).

Although the difference between the moderate and severe CKD groups was not significant, the elevation in Group 3 compared to the control group was statistically significant (*p* = 0.030). This observation points to a potential link between altered ET-2 regulation and the presence of advanced kidney damage [25,26,27]. Elevated levels of ET-2 expression have been identified in renal carcinoma cell lines, suggesting a potential role for ET-2 in renal damage [28]. Conversely, ET-1 levels showed neither a comparable consistent trend nor statistically significant differences with the progression of renal disease. The Mdn ET-1 levels were 37.57 pg/mL in controls, 44.32 pg/mL in the moderate CKD group, and 37.89 pg/mL in the severe CKD group (*p* > 0.05 for all comparisons). This differential behavior reinforces the hypothesis that ET-2 may play a distinct pathophysiological role in CKD. While ET-2 levels consistently increase with worsening eGFR, ET-1 appears to be a more general marker of renal damage. Elevated ET-1 levels in CKD are most often interpreted as a reflection of systemic vascular complications, including hypertension, atherosclerosis, and heart failure, which are prevalent in advanced stages of the disease [29,30,31]. The disparity in the behavior of ET-2 and ET-1 can be explained by their differential sites of production and regulatory mechanisms. While ET-1 is the most abundant and potent isoform, primarily produced by vascular endothelium, ET-2 is synthesized predominantly in the kidney and the gut [8,9,25,32].

The localized production of ET-2 within the kidneys suggests that its circulating levels may more directly reflect disrupted regulation and local pathological processes in renal tissue during the progression of CKD. The observed increase in circulating ET-2 levels with worsening renal function may represent a biologically plausible consequence of increased local synthesis, impaired renal clearance, or a combination of both; however, direct evidence for ET-2 renal clearance in humans is currently lacking [26,27]. In this context, ET-2 may serve as a more specific and sensitive biomarker for monitoring local renal changes in CKD compared to ET-1, whose levels are more likely to reflect systemic vascular disease and associated complications [8,25,26]. ET-1 is widely synthesized throughout the body and is associated with generalized endothelial dysfunction, whereas ET-2 is more specifically expressed in the kidneys and may reflect local changes [32,33]. Elevated ET-2 levels in CKD could result from dysregulated local synthesis or impaired clearance, both of which are linked to renal dysfunction [8,33].

### 3.2. Proposed Role of ET-2 in CKD Progression

Although ET-1 is the most extensively studied endothelin isoform in CKD [8,9], the present study highlights a potentially distinct role for ET-2 in the context of renal dysfunction and systemic inflammation. In our cohort, ET-2 concentrations were significantly higher in patients with advanced CKD and in subgroups characterized by elevated inflammatory markers (hsCRP and IL-6), whereas ET-1 and ET-3 did not show comparable group-wise differences. These findings suggest that ET-2 may be more closely linked to inflammatory and metabolic disturbances accompanying CKD progression than other endothelin isoforms.

The endothelin system is known to influence renal hemodynamics, inflammation, and fibrotic remodeling primarily through activation of ETA and ETB receptors, which are widely expressed in renal vascular, tubular, and interstitial cells [8,34]. ET-1-mediated ETA activation has been consistently associated with vasoconstriction, inflammation, and fibrosis, contributing to CKD progression [8,35,36,37]. In contrast, the biological role of ET-2 in renal disease remains less clearly defined. Our observation that ET-2 levels were associated with markers of systemic inflammation, rather than with purely hemodynamic parameters, suggests that ET-2 may reflect inflammatory signaling pathways rather than classical vasoregulatory mechanisms. Similar associations between endothelins and inflammatory activity have been reported in experimental and clinical studies [11,38].

Despite the high structural similarity between ET-1 and ET-2, differing by only two amino acids [32], accumulating evidence indicates that their biological roles are not identical. Experimental models have demonstrated that global deletion of the ET-2 gene results in phenotypes distinct from those observed in ET-1- or ET-3-deficient animals, supporting the concept that ET-2 functions as a distinct signaling entity rather than a simple surrogate of ET-1 [32]. In this context, the associations observed in our study between ET-2, inflammatory markers, and CKD severity are consistent with emerging data implicating ET-2 in inflammatory and tissue-specific regulatory processes [8,11].

Notably, the present study does not provide direct mechanistic evidence regarding the intracellular pathways through which ET-2 exerts its effects in the kidney. Experimental studies have established the TGF-β–SMAD signaling pathway as a central mechanism driving fibrogenesis across multiple organs, including the kidney [39,40,41]. However, whether ET-2 directly interacts with or modulates these profibrotic pathways in renal cells remains speculative and has not yet been experimentally demonstrated. Accordingly, the associations observed in the present study should be interpreted as clinical associations rather than evidence of causality.

Overall, our results underscore the importance of assessing individual endothelin isoforms rather than viewing the endothelin system as a uniform pathway. The distinct behavior of ET-2 in this study—particularly its links to inflammation and advanced CKD—highlights its promise as a biomarker of disease severity. Longitudinal and mechanistic studies are needed to elucidate ET-2‘s role in CKD pathophysiology and evaluate its potential as a therapeutic target.

### 3.3. The Connection Between ET-2, Uric Acid, and Systemic Inflammation

In addition to the link of ET-2 with worsening renal function, our results also reveal strong associations of ET-2 with hyperuricemia and systemic inflammation. Analysis of the entire cohort established a statistically significant positive correlation between ET-2 levels and UA (*rho* = 0.243, *p* = 0.036). The observed correlation between ET-2 and UA suggests that increased UA in progressing CKD may initiate a cascade of events, including oxidative stress and inflammation, which, in turn, leads to increased ET-2 production in the kidneys [42]. Thus, ET-2 may be not merely a marker, but an active participant in the pathophysiological process mediated by hyperuricemia. This correlation is further supported by the group comparison, where patients with elevated UA levels had significantly higher ET-2 concentrations (Mdn = 26.47 pg/mL) compared to those with normal levels (Mdn = 21.75 pg/mL, *p* = 0.015).

Although UA is known as a potent antioxidant [43], it can act as a pro-oxidant in certain microenvironments, such as renal tissue [43,44]. Under conditions of hyperuricemia, elevated UA levels can induce oxidative stress and inflammation through several mechanisms. These include reducing nitric oxide (NO) [45] bioavailability, activating NADPH oxidase [46,47], and stimulating pro-inflammatory signaling pathways such as NF-κB and MAPK could exacerbate systemic inflammation, potentially contributing to the progression of CKD and related complications [42,48]. Since ET-2 is produced by renal tissue [49], it is possible that under conditions of chronic inflammation and oxidative stress, renal cells increase ET-2 production as part of the local pathological response. This hypothesis is reinforced by the established positive associations between ET-2 levels and classic markers of systemic inflammation. ET-2 levels were significantly elevated in patients with high levels of hsCRP (Mdn = 26.40 pg/mL, *p* = 0.039) and IL-6 Mdn = 29.35 pg/mL, *p* = 0.038) compared to those with normal levels of the respective markers [50]. These data align with the well-established role of chronic inflammation as a key driver of renal progression [51,52]. Chronic inflammation in CKD results from the activation of resistant renal cells that produce pro-inflammatory cytokines and mediators [52,53].

A particularly interesting finding is the positive correlation between ET-2 and complement component C4 (rho = 0.373, *p* = 0.039) in Group 2 (patients with moderate renal dysfunction). This unique relationship, observed only at this specific stage of the disease, suggests that the role of ET-2 may be more nuanced and specifically linked to the activation of certain immunological pathways, such as the classical or lectin complement pathways. In contrast, ET-1 showed a negative correlation with C4 in Group 1 and Group 3, which may reflect a completely different pathophysiological mechanism. These specific correlations underscore the complexity of the endothelin pathways in CKD and open new avenues for research into the mechanisms by which ET-2 and ET-1 are modulated by various immunological and inflammatory pathways, including the complement system, during the course of renal disease. It is known that complement activation, particularly of components C3 and C4, has been implicated in the inflammatory processes that exacerbate renal injury, which is observed in conditions such as glomerulonephritis and chronic inflammation associated with CKD [54,55]. This intricate interplay between endothelins and the complement system suggests further investigation into their combined effects could yield valuable insights into the progression of CKD.

### 3.4. Study Limitations

Several limitations of the present study should be acknowledged. The lack of age-matched controls and the absence of age-adjusted statistical analyses may have introduced residual confounding. Although this reflects the real-world epidemiology of chronic kidney disease, which predominantly affects older individuals, the potential influence of age on circulating endothelin levels cannot be completely excluded. The cross-sectional design of the study precludes conclusions regarding temporal relationships or causality between endothelin-2 levels and CKD progression. In addition, concomitant pharmacological therapies were not included as adjustment variables in the statistical analyses, and their potential impact on endothelin, uric acid, and inflammatory marker levels cannot be excluded. The relatively modest sample size and the single-center design may limit the generalizability of the findings and warrant confirmation in larger, multicenter cohorts.

## 4. Materials and Methods

### 4.1. Study Design and Participants

This cross-sectional observational study included a total of 76 participants, comprising 64 patients diagnosed with CKD and 12 healthy control subjects. Disease stage was determined based on the estimated eGFR, calculated using the Modification of Diet in Renal Disease (MDRD) equation [56].

Participants were stratified into three groups according to kidney function. Group 1 (control group) consisted of 12 clinically healthy individuals with an eGFR ≥ 90 mL/min/1.73 m^2^. Group 2 (CKD stages 2 and 3a) included 32 individuals with an eGFR between 45 and 89 mL/min/1.73 m^2^, while Group 3 (CKD stages 3b and 4) comprised 32 individuals with an eGFR between 15 and 44 mL/min/1.73 m^2^. Patients with end-stage renal disease (stage 5), acute infections, autoimmune diseases, malignancies, recent surgery, or other inflammatory conditions that could interfere with biomarker interpretation were excluded. Baseline clinical and laboratory characteristics of the study participants are summarized in Table 4.

Additionally, endothelin levels (ET-1, ET-2, and ET-3) were further analyzed by stratifying the study population into subgroups based on normal or elevated levels of uric acid (UA), hsCRP, and IL-6. For UA analysis, comparisons were performed using sex-specific reference ranges.

### 4.2. Biochemical and Immunological Parameters

Blood samples were collected from all participants following an overnight fast. A broad panel of laboratory parameters was assessed, including markers of renal function, mineral metabolism, inflammation, and complement activation, alongside serum concentrations of endothelins.

#### 4.2.1. Renal Function Indicators

Serum creatinine (SCr) was measured using the Creatinine Jaffe Gen.2 kit (catalog number: 04810716190) on the Cobas C 311 analyzer. eGFR was calculated using the MDRD equation [56].

#### 4.2.2. Biochemical and Metabolic Markers

Serum Ca was measured using the Calcium Gen.2 kit (catalog number: 05061482190) on the Cobas C 311 analyzer.

Inorganic phosphate (Pi) was measured using the inorganic Phosphate Gen.2 kit (catalog number: 03183793122) on the Cobas C 311 analyzer.

Serum UA levels were measured using the Uric Acid Gen.2 kit (catalog number: 03183807190; Roche Diagnostics GmbH, Basel, Switzerland) on a Cobas C 311 analyzer. According to the manufacturer’s reference ranges provided in the assay instructions for the Uric Acid Gen.2 assay, normal serum UA levels were defined as 202–416 µmol/L for men and 143–339 µmol/L for women.

Hyperuricemia was defined as serum UA concentrations > 416 µmol/L in men and >339 µmol/L in women. No serum UA values below the reference range were observed in either patients or control subjects. For subgroup analyses, participants were classified into two groups: normal UA (within the reference range) and elevated UA (above the upper reference limit).

#### 4.2.3. Inflammatory Markers

Levels of the hsCRP were measured using the Cardiac C-Reactive Protein (Latex) High Sensitive (catalog number: 04628918190, Basel, Switzerland: Roche Diagnostics GmbH). The analyses were performed on a Cobas C 311 analyzer through a turbidimetric immunoassay, following the manufacturer’s instructions. Based on these measurements, patients were divided into a group with normal hsCRP levels (<3.0 mg/L) and a group with elevated hsCRP levels (≥3.0 mg/L). These thresholds are commonly used in clinical practice to identify individuals with low inflammatory risk versus elevated inflammatory risk, as defined by guidelines from the American Heart Association and Centers for Disease Control and Prevention [57].

IL-6 levels in the participants were quantitatively determined using an electrochemiluminescence immunoassay (ECLIA) with a commercially available Elecsys^®^ IL-6 kit (catalog number: 09015604190; Roche Diagnostics GmbH, Basel, Switzerland). The analyses were performed on an automated immunoassay analyzer Cobas E 411 (Roche Diagnostics), following the manufacturer’s instructions. Based on these measurements, patients were divided into a group with normal IL-6 levels (<7.0 pg/mL) and a group with elevated IL-6 levels (≥7.0 pg/mL). These thresholds are consistent with reference values established for healthy adult populations using this specific assay, as indicated in the manufacturer’s insert for the Elecsys^®^ IL-6 kit (Roche Diagnostics GmbH, Basel, Switzerland). The lower limit of quantification for the IL-6 assay was 1.5 pg/mL; values below this threshold were reported by the analyzer as 1.50 pg/mL.

Complement factor C3 was measured using the Tina-quant Complement C3c kit (catalog number: 03001938322) on the Cobas C 311 analyzer using an immunoturbidimetric assay.

Complement factor C4 was measured using the Tina-quant Complement C4 kit (catalog number: 03001962322) on the Cobas C 311 analyzer using an immunoturbidimetric assay.

#### 4.2.4. Endocrine Markers

Parathyroid hormone (PTH) was measured using the Elecsys PTH kit (catalog number: 11972103122) on the Cobas E 411 analyzer, utilizing an electrochemiluminescence immunoassay (ECLIA) method.

#### 4.2.5. Endothelin Levels

Serum levels of endothelin-1 (ET-1), endothelin-2 (ET-2), and endothelin-3 (ET-3) were quantified using commercially available enzyme-linked immunosorbent assay (ELISA) kits from FineTest^®^ (Wuhan, China). The specific kits used were: Endothelin-1 (EDN1): Human EDN1 (Endothelin-1) ELISA Kit (Catalog No.: EH0648, Revision: V4.0); Endothelin-2 (EDN2): Human EDN2 (Endothelin-2) ELISA Kit (Catalog No.: EH2114, Revision: V4.0); and Endothelin-3 (EDN3): Human EDN3 (Endothelin-3) ELISA Kit (Catalog No.: EH2113, Revision: V4.0). All assays were performed according to the manufacturers’ instructions. The absorbance was measured at 450 nm using an automatic micro-ELISA plate reader (Coulter Microplate Reader UV Max, Molecular Devices Corp., Menlo Park, CA, USA).

### 4.3. Statistical Analysis

Descriptive statistics were used to summarize patient characteristics and biochemical results. Continuous variables were expressed as means ± standard deviation (SD) or Mdn with IQR, depending on data distribution. Categorical variables were presented as counts and percentages. All statistical analyses were performed using GraphPad Software (GraphPad Prism 8, San Diego, CA: GraphPad Software). Prior to conducting group comparisons or correlation analyses, the distribution of continuous variables was assessed using the Shapiro–Wilk test. As most variables did not follow a normal distribution, non-parametric statistical methods were applied. For comparisons of biochemical and inflammatory markers between the groups, the Mann–Whitney U test was used for pairwise group comparisons. Results are presented as Mdn and IQR.

For the continuous variables that followed a normal distribution (parametric data), comparisons between the groups were performed using the independent-samples *t*-test.

To examine potential associations between endothelin levels and other clinical or laboratory parameters, Spearman’s rank correlation coefficient (rho) was used. A *p*-value < 0.05 was considered statistically significant.

For descriptive purposes, baseline clinical and laboratory characteristics are presented as mean ± SD in Table 4. For inferential analyses and between-group comparisons, variables that did not follow a normal distribution were analyzed using non-parametric methods and are presented as medians with IQR in Table 1.

### 4.4. Ethical Considerations

The study was approved by the Research Ethics Committee of the Medical University–Pleven (IRB approval No. 797-REC/21 June 2024) and conducted in accordance with the principles of the Declaration of Helsinki. Written informed consent was obtained from all participants, and all data were handled confidentially and anonymously.

## 5. Conclusions

The current study is one of the few to examine the differential role of endothelin isoforms in CKD. Our data demonstrate that, unlike ET-1, ET-2 levels tend to rise in parallel with the deterioration of renal function. Furthermore, we established statistically significant positive correlations between ET-2 and the main inflammatory and metabolic markers such as UA, hsCRP, and IL-6. These findings suggest that ET-2 may function as a specific biomarker reflecting not only the degree of renal dysfunction but also the underlying inflammatory and metabolic pathways contributing to its progression. This positions ET-2 as a potential candidate for future research and clinical application in the diagnosis and monitoring of CKD, opening new perspectives for a deeper understanding and management of this complex disease.

## Figures and Tables

**Table 1:** Pairwise comparisons of clinical and biochemical variables between the three groups using Mann–Whitney U test. Parameters presented as mean ± standard deviation are marked with an asterisk (*) and were determined to be normally distributed based on Shapiro–Wilk test.

Variable	Total (n = 76)	Group 1 (n = 12)	Group 2 (n = 32)	Group 3 (n = 32)	Group Comparison	*U*	*Z*	*p*
	Mdn (IQR)	Mdn (IQR)	Mdn (IQR)	Mdn (IQR)				
Age (years)	66.00 (20.0)	44.50 (23.3)	69.00 (12.0)	67.50 (18.0)	G1-G2	21.5	−4.458	<0.001
					G1-G3	41	−3.981	<0.001
					G2-G3	470	−0.358	0.721
eGFR (mL/min/1.73 m^2^)	50.00 (43.0)	93.50 (28.5)	58.00 (18.0)	24.00 (22.5)	G1-G2	25	−4.362	<0.001
					G1-G3	0	−5.063	<0.001
					G2-G3	0	−6.822	<0.001
SCr (μmol/L)	115.00 (85.0)	72.50 (18.5)	96.00 (29.0)	224.00 (172.5)	G1-G2	48	−3.738	<0.001
					G1-G3	0	−5.061	<0.001
					G2-G3	10	−6.683	<0.001
UA (μmol/L)	333.00 (87.0)	264.50 (144.3)	329.00 (80.0)	348.50 (83.8)	G1-G2	120.5	−1.774	0.076
					G1-G3	103	−2.345	0.018
					G2-G3	412	−1.155	0.248
Ca (mmol/L)	2.37 (0.16)	2.40 (0.17)	2.41 (0.13)	2.33 (0.16)	G1-G2	149.5	−0.99	0.328
					G1-G3	120.5	−1.885	0.059
					G2-G3	370	−1.734	0.083
Pi (mmol/L) *	1.23 ± 0.311	1.10 ± 0.170	1.303 ± 0.254	1.35 ± 0.397	G1-G2	-	-	0.367
					G1-G3	-	-	0.006
					G2-G3	-	-	0.018
PTH (pg/mL)	33.11 (29.24)	24.12 (15.19)	28.48 (26.20)	50.68 (94.13)	G1-G2	133	−1.435	0.157
					G1-G3	49	−3.768	<0.001
					G2-G3	214	−3.877	<0.001
hsCRP (mg/L)	2.93 (4.11)	1.68 (1.35)	3.11 (3.26)	3.29 (7.64)	G1-G2	93	−2.518	0.011
					G1-G3	87.5	−2.754	0.005
					G2-G3	456	−0.55	0.582
IL-6 (pg/mL)	1.50 (3.27)	1.50 (0.00)	1.50 (1.95)	2.89 (8.92)	G1-G2	130	−1.821	0.134
					G1-G3	103.5	−2.548	0.018
					G2-G3	363	−1.948	0.051
C3 * (g/L)	1.36 (0.29)	1.20 (0.47)	1.44 (0.32)	1.32 (0.33)	G1-G2	-	-	0.081
					G1-G3	-	-	0.813
					G2-G3	-	-	0.005
C4 (g/L)	0.27 (0.09)	0.23 (0.10)	0.29 (0.12)	0.27 (0.08)	G1-G2	101	−2.306	0.021
					G1-G3	121.5	−1.864	0.063
					G2-G3	439.5	−0.779	0.436
ET-1 (pg/mL)	41.30 (26.14)	37.57 (30.17)	44.32 (26.39)	37.89 (23.46)	G1-G2	143.5	−1.278	0.204
					G1-G3	172	−0.527	0.612
					G2-G3	424.5	−1.175	0.24
ET-2 (pg/mL)	24.14 (10.26)	19.32 (14.58)	25.15 (10.54)	24.49 (8.62)	G1-G2	122.5	−1.832	0.067
					G1-G3	110.5	−2.148	0.030
					G2-G3	491	−0.282	0.778
ET-3 (pg/mL)	23.95 (15.39)	24.35 (27.69)	25.43 (16.14)	22.90 (13.50)	G1-G2	192	0	1.000
					G1-G3	149.5	−1.12	0.267
					G2-G3	425	−1.168	0.243

Abbreviations: eGFR: estimated glomerular filtration rate; SCr: serum creatinine; UA: uric acid; ca: calcium; Pi: inorganic phosphate; PTH: parathyroid hormone; hsCRP, high-sensitivity C-reactive protein; IL-6: interleukin-6; C3: complement component 3; C4: complement component 4; ET-1: endothelin-1; ET-2: endothelin-2; ET-3: endothelin-3.

**Table 2:** Spearman’s rho correlations in the total cohort.

Variable 1	Variable 2	Correlation Coefficient (Spearman’s Rho)	*p*-Value
Age	eGFR	−0.294	0.010
Age	UA	0.254	0.028
Age	C4	0.232	0.045
Age	ET-3	−0.238	0.040
Gender	SCr	−0.370	<0.001
Gender	Ca	0.275	0.017
eGFR	SCr	−0.956	<0.001
eGFR	UA	−0.378	<0.001
eGFR	Ca	0.237	0.041
eGFR	Pi	−0.389	<0.001
eGFR	PTH	−0.571	<0.001
eGFR	hsCRP	−0.323	0.005
eGFR	IL-6	−0.381	<0.001
SCr	UA	0.379	<0.001
SCr	Ca	−0.303	0.008
SCr	Pi	0.307	0.007
SCr	PTH	0.529	<0.001
SCr	hsCRP	0.270	0.019
SCr	IL-6	0.336	0.003
UA	hsCRP	0.236	0.041
UA	ET-2	0.243	0.036
UA	Pi	0.298	0.009
Ca	PTH	−0.286	0.013
Ca	C3	0.330	0.004
Pi	C4	0.238	0.040
PTH	hsCRP	0.366	<0.001
PTH	IL-6	0.331	0.004
hsCRP	IL-6	0.612	<0.001
hsCRP	C3	0.378	<0.001
hsCRP	C4	0.615	<0.001
IL-6	C4	0.278	0.016
C3	C4	0.462	<0.001
C4	ET-1	−0.409	<0.001
ET-1	ET-3	0.316	0.005

Abbreviations: eGFR: estimated glomerular filtration rate; SCr: serum creatinine; UA: uric acid; Ca: calcium; Pi: inorganic phosphate; PTH: parathyroid hormone; hsCRP, high-sensitivity C-reactive protein; IL-6: interleukin-6; C3: complement component 3; C4: complement component 4; ET-1: endothelin-1; ET-2: endothelin-2; ET-3: endothelin-3.

**Table 3:** Significant correlations (Spearman’s rho) in Group 2 and Group 3.

Group	Variable 1	Variable 2	Correlation Coefficient (Spearman’s Rho)	*p*-Value
2	Gender	SCr	−0.718	<0.001
	Gender	Ca	0.378	0.036
	Gender	Pi	0.475	0.007
	PTH	ET-1	−0.367	0.042
	hsCRP	IL-6	0.635	<0.001
	hsCRP	C3	0.610	<0.001
	hsCRP	C4	0.586	<0.001
	IL-6	C3	0.558	<0.001
	C3	C4	0.514	0.003
	C4	ET-1	−0.432	0.015
	C4	ET-2	0.373	0.039
3	Gender	ET-2	0.435	0.013
	eGFR	SCr	−0.925	<0.001
	eGFR	Pi	−0.656	<0.001
	eGFR	PTH	−0.474	0.006
	eGFR	C4	−0.377	0.033
	SCr	Pi	0.629	<0.001
	SCr	PTH	0.392	0.027
	UA	Pi	0.434	0.013
	Ca	PTH	−0.373	0.036
	Ca	C3	0.365	0.040
	Ca	ET-3	−0.389	0.028
	Pi	C4	0.485	0.005
	PTH	hsCRP	0.508	0.003
	PTH	IL-6	0.392	0.027
	hsCRP	IL-6	0.723	<0.001
	hsCRP	C4	0.696	<0.001
	IL-6	C4	0.412	0.019
	C4	ET-1	−0.586	<0.001
	ET-1	ET-3	0.519	0.002

Abbreviations: eGFR: estimated glomerular filtration rate; SCr: serum creatinine; UA: uric acid; Ca: calcium; Pi: inorganic phosphate; PTH: parathyroid hormone; hsCRP, high-sensitivity C-reactive protein; IL-6: interleukin-6; C3: complement component 3; C4: complement component 4; ET-1: endothelin-1; ET-2: endothelin-2; ET-3: endothelin-3.

**Table 4:** Clinical and laboratory characteristics of the studied groups.

	Group 1 (n = 12) 5♀/7♂		Group 2 (n = 32) 17♀/15♂		Group 3 (n = 32) 21♀/11♂	
**Etiology of CKD**						
			***n***	**(%)**	***n***	**(%)**
Hypertensive nephropathy			12	37.5	11	34.375
Diabetic nephropathy			9	28.125	10	31.25
Chronic pyelonephritis			4	12.5	5	15.625
Chronic Glomerulonephritis			4	12.5	4	12.5
Chronic interstitial nephritis			2	6.25	1	3.125
Polycystic kidney disease			1	3.125	1	3.125
**Concomitant Pharmacological Therapy**						
			***n***	**(%)**	***n***	**(%)**
Diuretics			15	46.875	26	81.25
ACE inhibitors			8	25	13	40.625
Angiotensin receptor blockers			10	31.25	11	34.375
SGLT2 Inhibitors			14	43.75	25	78.125
Statins			18	56.25	18	56.25
Urate-lowering therapy			14	43.75	21	65.625
Antiplatelet and anticoagulant therapy			12	37.5	15	46.875
Corticosteroids			2	6.25	1	3.125
**Clinical and Laboratory Characteristics**						
	**Mean**	**SD**	**Mean**	**SD**	**Mean**	**SD**
Age (years)	42.33	13.75	67.84	8.62	65.59	12.42
eGFR (mL/min/1.73 m^2^)	93.92	21.31	61.39	11.96	25.78	12.43
SCr (μmol/L)	69.50	14.64	97.42	20.35	273.13	174.54
UA (μmol/L)	276.50	89.51	331.77	75.84	352.31	75.38
Ca (mmol/L)	2.43	0.11	2.39	0.09	2.33	0.16
Pi (mmol/L)	1.10	0.17	1.16	0.19	1.35	0.40
PTH (pg/mL)	23.69	12.68	32.44	15.12	96.87	111.04
hsCRP (mg/L)	2.79	3.73	7.06	12.13	12.88	31.17
IL-6 (pg/mL)	5.97	15.48	3.41	3.81	8.21	11.24
C3 (g/L)	1.30	0.25	1.45	0.23	1.28	0.21
C4 (g/L)	0.23	0.05	0.29	0.08	0.27	0.08
ET-1 pg/mL	38.80	15.87	51.93	31.41	44.45	21.75
ET-2 pg/mL	22.66	10.99	26.43	7.22	32.26	26.55
ET-3 pg/mL	33.36	18.76	34.62	28.86	27.03	14.64

Abbreviations: eGFR: estimated glomerular filtration rate; SCr: serum creatinine; UA: uric acid; Ca: calcium; Pi: inorganic phosphate; PTH: parathyroid hormone; hsCRP, high-sensitivity C-reactive protein; IL-6: interleukin-6; C3: complement component 3; C4: complement component 4; ET-1: endothelin-1; ET-2: endothelin-2; ET-3: endothelin-3; ACE: angiotensin-converting enzyme; SGLT2: sodium–glucose transport 2. Values are presented as mean ± SD for descriptive purposes.

## Data Availability

The original contributions presented in this study are included in the article. Further inquiries can be directed to the corresponding authors.

## Abbreviations

The following abbreviations are used in this manuscript:

C3	Complement Component 3
C4	Complement Component 4
Ca	Calcium
CKD	Chronic Kidney Disease
ECLIA	Electrochemiluminescence Immunoassay
eGFR	Estimated Glomerular Filtration Rate
ELISA	Enzyme-Linked Immunosorbent Assay
ET-1	Endothelin-1
ET-2	Endothelin-2
ET-3	Endothelin-3
hsCRP	High-Sensitivity C-Reactive Protein
IL-6	Interleukin-6
IQR	Interquartile Range
MDRD	Modification of Diet in Renal Disease
Mdn	Median
Pi	Inorganic Phosphate
PTH	Parathyroid Hormone
SCr	Serum Creatinine
SD	Standard Deviation
UA	Uric Acid
